# Contribution of Immunoglobulin Enhancers to B Cell Nuclear Organization

**DOI:** 10.3389/fimmu.2022.877930

**Published:** 2022-06-24

**Authors:** Charlotte Bruzeau, Jeanne Cook-Moreau, Eric Pinaud, Sandrine Le Noir

**Affiliations:** UMR CNRS 7276, INSERM 1262 and Université de Limoges: Contrôle de la Réponse Immune B et des Lymphoproliférations, 2 Rue du Pr. Descottes, Limoges, France

**Keywords:** immunoglobuline genes, enhancers, nuclear organization, B lymphocytes, chromatin loops

## Abstract

B cells undergo genetic rearrangements at immunoglobulin gene (*Ig*) loci during B cell maturation. First *V(D)J* recombination occurs during early B cell stages followed by class switch recombination (CSR) and somatic hypermutation (SHM) which occur during mature B cell stages. Given that RAG1/2 induces DNA double strand breaks (DSBs) during *V(D)J* recombination and AID (Activation-Induced Deaminase) leads to DNA modifications (mutations during SHM or DNA DSBs during CSR), it is mandatory that *IgH* rearrangements be tightly regulated to avoid any mutations or translocations within oncogenes. Ig loci contain various *cis*-regulatory elements that are involved in germline transcription, chromatin modifications or RAG/AID recruitment. *Ig cis*-regulatory elements are increasingly recognized as being involved in nuclear positioning, heterochromatin addressing and chromosome loop regulation. In this review, we examined multiple data showing the critical interest of studying *Ig* gene regulation at the whole nucleus scale. In this context, we highlighted the essential function of *Ig* gene regulatory elements that now have to be considered as nuclear organizers in B lymphocytes.

## Introduction

**Figure 1:**
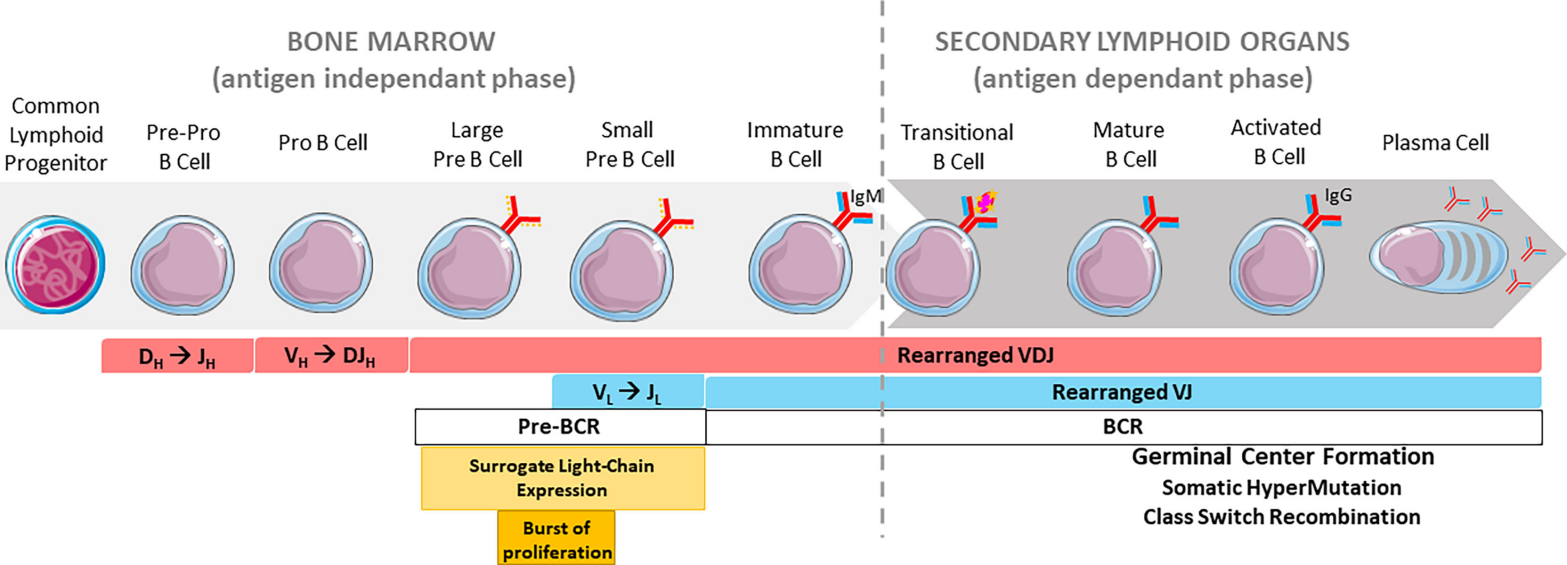
Overview of B cell development. Chronological order of B cell stages in the bone marrow (left) and secondary lymphoid organs (right). The pre-BCR is represented by a continuous red line and a discontinuous orange line (surrogate light chain). The BCR is represented by continuous red and blue lines.

To produce highly specific antibodies, B cells undergo genetic modifications of their immunoglobulin (*Ig*) genes. Among these events, *V(D)J* recombination takes place in the bone marrow during the early steps of B cell development and occurs in an antigen-independent manner. Mature B cells migrate towards secondary lymphoid organs and continue their differentiation once stimulated by antigens. This process integrates secondary beneficial DNA remodeling events including class switch recombination (CSR) and somatic hypermutation (SHM) but can also induce B cell death through locus suicide recombination (LSR), a detrimental rearrangement that abrogates surface B cell receptor expression (1). These events, all mediated by the activation-induced deaminase (AID) enzyme, characterize the late antigen-dependent phase of developing cells (**Figure 1**).

Nuclear organization and chromosome distribution in B-lineage cells have to be considered as important parameters for the control of all these events. Chromosomes are not randomly scattered in the nucleus; their positions change in a dynamic fashion especially during the B cell lifespan. Indeed, distinct organizational levels co-exist in the genome on nucleosomal (genes and loci), supranucleosomal (chromatin domains and compartments) and nuclear (chromosome territories) scales (2–4). In addition to histone mark patterns that reflect the nucleosomal context, the emergence of new molecular biology approaches led to the discovery of Topologically Associated Domains (TADs) and the dynamic loop extrusion model (5). Such methods, based on chromosome capture conformation (6) (3C, 4C and Hi-C), make it possible to evaluate additional levels of gene regulation at the supranucleosomal scale. This particular level of nuclear topology includes TAD structures, A and B chromatin compartments, DNA loops and interchromatin space. Gene transcription takes place in the A-euchromatin compartment whereas B-heterochromatin prevents it. In a simplified scheme, A and B compartments are respectively positioned at the center of nuclei and at the nuclear periphery (4). More recently, the Volk’s group showed that heterochromatin and euchromatin, respectively defined as B and A compartments, are localized at the nucleus periphery, leaving the nucleus center devoid of nucleosomes (7). As a structural unit of genome organization, a TAD is a large chromosomal region in which the contact frequencies between genes or regulatory regions are higher than elsewhere in the genome. TADs themselves are subdivided into multiple sub-TAD structures (chromatin loops) that undergo dynamic cell-type specific connections. The mouse genome contains around 2000 TADs, each with an approximate mean size of 1 megabase (Mb) (8). Indeed, some dynamic processes drive chromatin regions into a free space termed the interchromatin compartment in order to permit gene segment interactions. Such contacts between gene portions occur either in active (A) or inactive (B) chromatin compartments and it is widely recognized that interactions take place within the same TAD. Among chromatin compartments, long-range homologous contacts (A–A or B–B) are largely favored over heterologous contacts (A–B) (9). Moreover, additional TAD interactions exist since chromosome portions are not only able to establish close contacts in *cis*, but also in *trans* with other chromosomes (10). Some of these *trans* interactions have been documented in the case of olfactory receptor (11) and Th2 cytokines genes (12). At TAD extremities, TAD borders are enriched in CTCF (CCCTC-binding Factor) insulator protein, mediator complexes (MED1, MED12) as well as active histone marks (H3K4me3 and H3K36me3) (8). TAD borders display specific “insulating” features, preventing loci located on each side of this border to establish contacts (8).

By considering genome nuclear topology, these emerging models are particularly relevant for the tightly-regulated *Ig* gene loci. Most *Ig* gene regulation studies have so far been performed at the nucleosomal scale (epigenetic modifications and regulatory transcription of loci and gene segments). The increasing interest in understanding gene regulation at the whole nucleus scale prompted B cell scientists to revisit previous models at both supranucleosomal (DNA loops and TADs) and nuclear (chromosome territories and nuclear position) levels (13–22). To provide a clearer picture of how B cell development is tightly regulated by the nuclear location of *Ig* genes, including chromosome looping and loci positioning in the mouse, we will begin with an overview of *Ig* genes and their enhancers and then focus on the role of their main enhancers on 3D-nuclear organization. The relationship between *Ig* and respective enhancers will be discussed in this review.

## Overview of Immunoglobulin Genes and Their Regulatory Elements

In mice, *Ig* genes are encoded by three loci located on three distinct chromosomes. The immunoglobulin heavy chain (*IgH*) locus lies on chromosome 12. Immunoglobulin light (*IgL*) chain loci contain either kappa immunoglobulin light chain (*Ig*κ) or lambda immunoglobulin light chain (*Ig*λ) genes and are respectively located on chromosomes 6 and 16. In mice, at least 95% of B cells express Igκ light chains (13, 14).

### Immunoglobulin Heavy (IgH) Chain Loci

**Figure 2:**
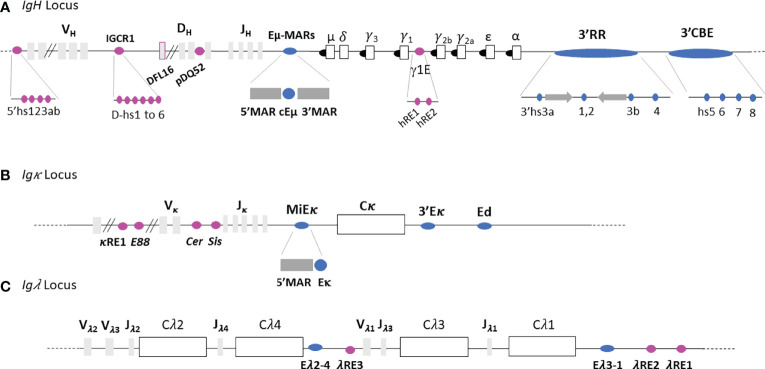
Immunoglobulin loci. **(A)** *IgH* locus. **(B)** *Igκ* locus. **(C)** *Igλ* locus. Light grey and white rectangles represent *V, D* and *J* gene segments and constant genes respectively. Black circles represent switch regions and blue and pink ovals represent respectively the main Ig loci regulatory elements and other enhancers. Not to scale.

The *IgH* locus spans approximately 3Mb, in its germline configuration, and contains various *cis*-regulatory regions (**Figure 2A**). From 5’ to 3’, the *5’hs123ab* elements, are situated upstream from the first *V_H_* segment (15). The intergenic *V_H_D_H_* region, located between the most distal *D_H_* segment, *DFL16*, and the most proximal *V_H_* segment, *V_H_*_7183a.2.3_, contains six DNase I sensitive sites (*hs1* to *6*) among which *hs4* and *hs5* are CTCF binding sites (17, 18). This set is also called Intergenic Control Region 1 (*IGCR1*) (16, 17). The promoter/enhancer *pDQ52*, is located just upstream from *DQ52* (18). The *Eµ-MARs* region, spanning about 1kb, is composed of a 220-base pair (bp) core enhancer element (*cEµ*) flanked by two matrix attachment regions (*MARs*) located between the last *J_H_* exon and the *Sµ* region. Between the *Cγ1* and *Cγ2b* constant genes, two transcriptional enhancers *hRE1* and *hRE2* (19) form the γ1E regulatory element (20). At the 3’extremity, the *3’Regulatory Region* (*3’RR*), spanning more than 30 kb, is composed of four enhancers, called *hs3a*, *hs1.2*, *hs3b* and *hs4 (* 21). The central *hs1.2* enhancer is flanked by inverted repeated intervening sequences (*IRIS*) that form a 25kb-long quasi-palindrome (22). Downstream from this palindrome, *hs4* is the most distal element harboring an enhancer activity within the *3’RR* (23). Although highly divergent in different species, *IRIS* sequences always stand as inverted copies on both sides of *hs1.2*, conserving a singular symmetry within the *3’RR* (24). Downstream from the *3’RR*, four *hs* elements (*hs5*, *hs6, hs7* and *hs8*) lie in a region containing ten CTCF Binding Elements (*3’CBEs*) (25, 26). This region acts as an insulator and delimits the *3’ IgH* TAD border (25, 26).

Our group and others developed numerous mouse models carrying deletions within the *IgH* regulatory elements that helped elucidate *Eµ* and *3’RR* super-enhancer functions during early and late B cell development (20, 27–37).

### Immunoglobulin Light Chain Loci: Igκ and Igλ

The 3.2 Mb *Ig*κ locus contains regulatory elements within the *Vκ* segments (**Figure 2B**) which are partially homologous to those within the *IgH*: *κRE1* (19) and *E88* (38). Six DNase I hypersensitive sites (hs), *hs1* to *6*, are located in the intervening *Vκ-Jκ* region (39, 40). *Hs1-2* forms *Cer* (Contracting element for recombination) and *hs3-6* comprises the *Sis* (Silencer in the intervening sequence) elements. A MAR-intronic Eκ enhancer (*MiEκ*) (41–43), located downstream from the last *Jκ* segment, is composed of a 5’flanking matrix attachment region (*MAR*κ) and an intronic enhancer. The *3’E*κ enhancer region is situated downstream from the unique *Cκ* gene (44). A third regulatory region, called *Ed* (due to its downstream location within the *Igκ* locus), is located distal to *3’Eκ* (45).

In comparison to *IgH* and *Ig*κ loci, the *Ig*λ locus is smaller (200kb) and uniquely organized. It comprises four families which contain a pair of *J*_λ_ and *C*_λ_ segments with only three *V*_λ_ segments (46) (**Figure 2C**). The *Ig*λ locus contains two main enhancers called *E_λ2-_*_4,_ located between *Cλ4* and *Vλ1*, and *E_λ3-1_* located downstream from *Cλ1* (47). Three supplementary elements featuring enhancer activity have been described: *λRE3*, *λRE2* and *λRE1* (19). *λRE3* lies between *E_λ2-4_* and Vλ1 while *λRE1* and *λRE2* are located close to the *E_λ3-1_* enhancer. Both *Ig*λ enhancers, *E_λ2-4_* and *E_λ3-1_* are involved in transcription and *VJ* rearrangement regulation (47). *λRE* elements, especially *λRE1* and *λRE3*, have been shown to potentiate the enhancer activity of *E_λ3-1_* and *E_λ2-4_*, in pro-B and plasma cells respectively (19).

### Immunoglobulin Joining Chain Loci: IgJ

Located on chromosome 5 in mice, the *IgJ* loci encodes the joining peptide (J) chain which promotes active IgA (48) or IgM (49) secretion by ensuring efficient assembly of these Ig subtypes in plasma cells.

## Nuclear Organization Driven by Ig Enhancers at Early B Cell Stages

Early B cell development takes place in bone marrow where a lymphoid precursor progresses through different stages to the final immature stage. This progression is concomitant with *V(D)J* recombination. Once engaged in the B cell lineage after expression of B cell specific transcription factors (50), the common lymphoid precursor (CLP) differentiates into a pre-pro B and then pro-B cell that undergoes *D_H_-J_H_* rearrangement at the *IgH* locus (51). Once the *DJ_H_* segment rearranges (52), the pro-B cell joins a *V_H_* segment to the previously rearranged *DJ_H_* segment and progresses to the large pre-B cell stage. At this stage, the *IgH* locus is completely rearranged and the cell expresses a pre-B Cell Receptor (pre-BCR) at its surface. The pre-BCR, indispensable for B cell development (53), is composed of a functional IgH chain linked to an invariant surrogate light chain, altogether associated with the Igα-Igβ transmembrane heterodimer signaling component. Pre-BCR signaling stops *IgH* rearrangement and triggers a burst of proliferation leading to the small pre-B cell stage and the occurrence of *V_L_-J_L_* rearrangements at *IgL* loci. Successful *IgL* chain rearrangement and production leads to membrane IgM expression and consequently a functional BCR (50), on the immature B cell. These cells then migrate towards secondary lymphoid organs and continue their maturation (**Figure 1**).

### IgH Sub-Nuclear Positioning and Chromatin Loops

**Figure 3:**
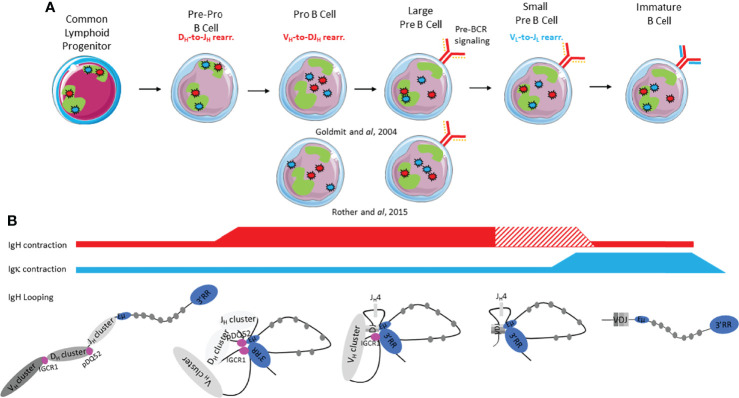
Immunoglobulin loci contraction, conformation, heterochromatin addressing and nuclear positioning during early B cell development. **(A)** *IgH* and *IgL* alleles are respectively represented by red stars and blue stars; the green form represents the pericentromeric heterochromatin compartment (PCH). The pre-BCR is represented by a continuous red line and a discontinuous orange line (surrogate light chain). The BCR is represented by continuous red and blue lines. **(B)** *Ig* loci contraction is shown by red and blue lines for *IgH* and *Igk* respectively: thickness of the line indicates degree of contraction. The hatched lines correspond to controversial studies.

*Ig* loci positioning within the nucleus is dynamic throughout early B cell development and it has been clearly shown that nuclear organization provides a critical level of regulation during *V(D)J* recombination, particularly for loci contraction/decontraction, nuclear positioning (center *vs* periphery) and heterochromatin addressing (**Figure 3**).

#### IgH Sub-Nuclear Positioning

Dynamic repositioning of the *IgH* locus throughout early stages is involved in *IgH* accessibility in order to correctly orchestrate *V(D)J* recombination (**Figure 3A**). In CLP and pre-pro-B cells, *IgH* loci are preferentially located at the nuclear periphery within a repressive compartment (54). While only few changes occur at immature stages (55), the pro-B cell stage undergoes, in contrast, global genome reorganization since around 20% of genes switch to A-B or B-A compartments (56). Indeed, both *IgH* alleles are relocated to the active A compartment to allow *D_H_-J_H_* recombination on both alleles. At the large pre-B cell stage, once *VDJ_H_* recombination completed, pre-BCR signaling induces repositioning of one *IgH* allele into a repressive centromeric domain. Although pericentromeric recruitment has been associated with allelic exclusion (57), this nuclear location does not preclude transcriptional expression of the non-productive allele (58). Monoallelic *IgH* addressing to pericentromeric heterochromatin (PCH) remains until the immature B cell stage (57).

#### IgH Locus Contraction and Chromatin Loops

Beyond nuclear positioning, *IgH* allele contraction/decontraction, as well as TAD and loop reorganization represent an additional level of regulation, considered as critical for early B cell development (59). At the pre-pro B cell stage, both *IgH* alleles are decontracted (60) and at the pro-B cell stage both *IgH* loci contract to bring *D_H_* et *J_H_* clusters closer to promote *D_H_* to *J_H_* rearrangement (**Figure 3B**). 3C (Chromosome Capture Conformation) and 4C (Circular Chromosome Capture Conformation) experiments revealed that *IgH* locus compaction was mediated by large loops in which *Eµ* interacts with *3’RR*, *IGCR1* and *V_H_* regions (5’ *V_H_7183* and 5’ *V_H_558*) (60). During the transition from pro-B to large pre-B cell stages, the entire *V_H_* region is brought into juxtaposition with *D_H_* elements due to extensive *IgH* locus contraction (60). This contraction is completed by looping within the *V_H_* region to facilitate *VDJ* recombination (57, 61). This builds a rosette-like structure and allows equal usage between proximal, middle and distal *V_H_* genes, essential for the generation of a diversified immunoglobulin repertoire (62). After productive *IgH* recombination and in response to pre-BCR signaling, locus contraction is reversed in small pre-B cells (60) or in large pre-B cells (57) depending on the studies. This decontracted state remains until the immature stage (57) Discrepancies between Rother’s (60) and Roldan’s (57) reports could be due to different probes used in 3D-FISH. Rother used three different probes encompassing distal *V_H_*, proximal *V_H_* and *C_H_* genes whereas Roldan employed only two probes including one spanning *Cγ1* and the other *V_H_J558* (distal). Ultimately, *IgH* looping plays a critical role in *V(D)J* recombination by forming a “recombination center” containing p*DQ52*, all four *J_H_* segments and *cEµ* (17). These loops permit RAG scanning between two recognition signal sequences (*RSS*) and therefore focal RAG targeting activity during *D_H_* to *J_H_* joining. This loop extrusion, is thought to ensure better sequence recognition by the RAG complex, avoiding off-targeting (63).

While *IgH* locus contraction and looping are important to permit equal usage of all *V_H_* genes, nuclear positioning of *IgH* loci seems more determinant than locus contraction in the orchestration of ordered *V(D)J* rearrangements (60).

### Igκ Nuclear Positioning and Chromosome Loops

In contrast to the *IgH* locus, the exact kinetics of *Igk* loci contraction and relocation within the nucleus remain unclear but it is admitted that dynamic changes also occur at *Igκ* loci. In CLP and pre-pro B cells, both *Igκ* alleles are located in PCH. At the pro-B cell stage, *Igk* loci relocate to a more central and active area within the nucleus (54, 64, 65). From large pre-B cells and until the immature stage, one *Igκ* allele becomes more closely associated with active chromatin and the other *Igκ* allele stays in the PCH (64) (**Figure 3A**). This chronology is still debated. Rother et al. described relocation of both *Igκ* loci only at pre-B cell stages (60). In small pre-B cells, contraction occurs at the *Igκ* locus, in preparation for *Vκ*-*Jκ* recombination and this contracted status persists until the immature B cell stage (39, 57) (**Figure 3B**). While some controversy still remains concerning contraction of *Igκ* locus in all early developmental stages, it is clear that redistribution of intra-loci interactions occurs at the small pre-B cell stage (60, 66). This redistribution, mediated by pre-BCR signaling, results in *Igκ* looping through ordered coordination between *MiEκ*, *3’Eκ* and *Sis* regulatory elements spread throughout the locus (67).

### Role of Ig Enhancers in B Cell Nuclear Organization

#### IgH V-D_H_ Intergenic Region and IGCR1

Beyond the observation that *IGCR1* interacts with the *3’CBEs* insulator region in pro-B cells (68, 69); recent studies suggest an insulation function for *IGCR1* itself. In an *IGCR1* deficient mouse model, interaction between the whole *V-D_H_* intergenic region and *3’CBEs* was abolished while *Eµ*/*3’RR* interaction was not (69). In *IGCR1* deficient pro-B cells, *Eµ*/*IGCR1* interaction was decreased as expected but *Eµ*/*V_H_81* and *Eµ*/*3’RR* interactions still occurred (70). Moreover, the recombination center, in which *DFL1.6*/*DQ52*/*Eµ*/*hs4* interact in a *wt* context, was disturbed in *IGCR1* deficient pro-B cells. The absence of *IGCR1* allows an alternative bigger loop to occur between *V_H_81X*/*DQ52*/*Eµ*/*hs4.* By including proximal *V_H_* segments in the former loop, this new chromosome conformation alters *V(D)J* repertoire by decreasing distal *V_H_* usage (70). According to the actual model, *IGCR1* seems to work as an insulator to delimit *Eμ* action/function. These findings are contradicted by Busslinger’s group who carried out 4C-Seq experiments in *IGCR1* deficient pro-B cells where interaction patterns remain unchanged compared to *Rag* ^-/-^ pro-B cells (71). This discrepancy between studies could be explained by the restriction enzymes used in 4C experiments. When Busslinger’s group used sequentially *HindIII* (6pb cutter) and *Sau3AI* enzymes, Sen’s group used two 4pb cutter enzymes (*MseI* and *NlaIII*) that generate smaller fragments and probably offer higher resolution (**Table 1**).

**Table 1:** Contribution of Immunoglobulin enhancers to B Cell Nuclear Organization.

	Enhancers	Early development	Late development
IgH locus	**5'hs123ab**		
	**IGCR1**	*VDH-3’CBEs interactions (17, 69). *Maintain recombination center in pro B cells (DFL1.6; DQ52; Eµ; hs4) (17, 69). *Formation of alternative larger loop between VH81X, DQ52, Eµ and hs4 (70).	
	**pDQ52**		
	**Eµ-MAR**	*Loop formation between DFL16.1, IGCR1, Eµ and 3’RR. (17, 69). *Maintain *IgH* loci at nuclear periphery in pro B cells (17, 69).	
	γ**1E**		
	**3'RR**		*Maintain proximity of both IgH loci (Le Noir et al., 2016) (86). *hs3b and hs4 enhancers required for interaction between 3’RR and Eµ regions (74).
	**3'CBE**		*Total 3'CBE region involved in interactions between Eµ and targeted Switch regions (25).
Igk locus	κ**RE1**	*Interaction between Vk/Jk with MiEk, 3'Ek and 3'Ed (38).	
	**E88**		
	**hs1-2=Cer**	*Igk locus contraction (40).	
	**hs3-6=Sis**	*IgH and *Igk* adressing to PCH in pre-B cells (39).	
	**MiE**κ	*Igk locus contraction (60) Maintain *Igk* in an active compartment (72).	
	**3'E**κ	*Maintain *Igk* in active compartment at pre B cell stage (65) *IgH decontraction, relocalization to PCH and *IgH-Igk* association (72).	*Maintain *IgH* , *Igk* and *IgJ* loci close to nuclear periphery in plasma cells (65).
	**3'Ed**		

In the absence of study depicting the role of Ig enhancers the boxes are empty. Bold correspond to the name of the enhancers.

#### IgH DQ52 Enhancer and Eµ Region

Many studies have contributed to the identification and unraveling of the role of *Ig* gene enhancers on 3D loci conformation during early B cell development proving that such regulatory elements are clearly involved in subnuclear positioning and chromosome looping (intra-TAD modification). To decipher the role of *pDQ52* and the *Eµ* enhancer, Guo and colleagues generated two mouse models (17): P^-^E^-^ (both *pDQ52*/*DQ52* and *Eµ* enhancers are deleted) and P^-^E^+^ (only promotor *pDQ52* and *DQ52* segments are deleted). Using 3D-FISH, authors demonstrated that the *Eμ* enhancer is required for *IgH* locus contraction in pro-B cells. Indeed, in P^-^E^-^, but not in the P^-^E^+^ model, the large loops between *V-D_H_* intergenic regions (*DFL16.1*, *IGCR1), Eµ* and the *3’RR* are drastically reduced underlining the important role of *Eµ* in loop formation. Moreover, ChIP-Seq experiments coupled with 3C experiments demonstrated that such large domain interactions are shaped by CTCF and cohesin proteins (68). In this way, CTCF-mediated *IgH* looping facilitates the generation of a diversified repertoire by juxtaposing distal *V_H_* to *DJ_H_* regions (71). It has also been shown that, in pro-B cells, *cEµ* deletion leads to *IgH* relocation in close proximity to the nuclear periphery (17). Taken together, these studies show that the *Eµ* region has a pivotal role during early *V(D)J* recombination by regulating both long range interactions and *IgH* sub-nuclear positioning. While *Eµ* seems to facilitate *IgH* loops in developing B cells, the presence of this enhancer is not strictly necessary since another study showed, by 4C-Seq, that several long-range interactions remain detectable in the absence of *Eµ (* 71) (**Table 1**).

#### Igκ E88

The *E88* element located in the intervening *V-Jκ* region also participates in the generation of a diversified Ig repertoire (38). Endowed with enhancer activity starting at pre-B cell stages, *E88* regulates long-range *Igκ* chromatin interactions and participates in sub-TAD determination of the Ig*κ* locus. *E88* deletion in pro-B cells disrupts interactions between *Vκ/Jκ* genes and three other known enhancers, especially *MiE*κ, but also to some extent *3’E*κ and *3’Ed (* 38). It is then reasonable to consider *E88* as a major hub of *Igk* locus interactions critical for regulation of *Igk* repertoire diversity.

#### Igk Cer and Sis Elements

While of critical interest to generate a diversified antibody repertoire of the Igκ chain (39, 40); *Cer* and *Sis* regulatory elements located within the *Igκ* locus are also involved in *Ig* loci PCH addressing. It is now established that *IgH* and *Igκ* loci are monoallelically repositioned into PCH in pre-B cells (72). In *Sis* ^-/-^ pre-B cells, both *IgH* and *Igk* alleles do not relocate to PCH. While *Igκ* locus contraction and looping still occur normally in the absence of this element, *Sis*-deficient B cells harbor a biased *Igκ* repertoire with increased proximal *Vκ* gene usage accompanied by decreased distal *Vκ* utilization. Taken together, these results show that the *Sis* element is required for *Igκ* (*cis*-acting) and *IgH* (*trans*-acting) monoallelic positioning within the nucleus and somehow promotes a diversified antibody repertoire (39). *Cer* deletion leads to a strong increase in proximal *Vκ* usage with decreased distal *Vκ* usage. *Cer* ^-/-^ pre-B cells show normal epigenetic marks throughout the *Igκ* locus but the compaction level is clearly decreased suggesting that *Cer* regulates *Igκ* locus contraction (40). As a whole, *Sis* seems to be more involved in nuclear *Ig* loci positioning and *Cer* in *Igκ* locus conformation. The idea that nuclear positioning, rather than loci contraction, promotes Ig gene recombination (60) is thereby questioned by the reciprocal functions of *Sis* and *Cer* elements in the *Igκ* locus.

#### Igk MiEκ Element

*Igκ* locus contraction, mediated by *MiEκ*, seems to occur at the pro-B cell stage and remains until the pre-B cell stage. This observation indicates that locus contraction is lineage, but not stage specific (60). In pro-B cells, *MiEκ* deletion both provokes positioning of both *Igκ* alleles to PCH and reduces physical distance between *IgH* and *Igκ* loci evidence of *Ig* loci crosstalk at this stage. In pre-B cells, *MiEκ* deletion enforces both *Igk* and *IgH* allele positioning to PCH (76). The current model proposes that *MiEk* is more implicated in *Ig* loci sub-nuclear organization than in locus looping.

#### 3’ Eκ Region

Involvement of *3’Eκ* in early rearrangement of the *Igκ* locus is clearly established. This enhancer region is necessary for *Igκ* germline transcription activation, which is a prerequisite for *Vκ-Jκ* recombination (77). In line with the previously observed transcription defect, deletion of the*3’Eκ* enhancer induces premature repositioning of *Igκ* into PCH at the pre-B cell stage. Moreover, according to Park and colleagues, in pro-B cells, *Igκ* seems to be distant from the *IgH* locus although both *IgH* and *Igκ* appear to be located at the nuclear periphery (65). More strikingly, in *3’Eκ* deficient mice, Skok and colleagues also documented multiple defects in *IgH* decontraction, relocalization to PCH and *IgH-Igκ* association (76). Altogether these results suggest that *IgH* decontraction is dependent on PCH repositioning and *Igκ-IgH* colocalization. This particular model highlights an unexpected *trans* – acting effect upon deletion of an *Ig* gene enhancer. This observation supposes strong crosstalk between *Ig* loci in developing B cells and supports the hypothesis that regulatory regions are involved in interactions between loci. In parallel, crosstalk between *Ig*κ and *IgH* is a little more frequent in pre-B cells than in pro-B cells and becomes almost inexistent at immature stages (65).

## Nuclear Organization Driven by Ig Enhancers in Late Developing B Cells

In secondary lymphoid organs, mature B cells can encounter soluble or membrane antigens able to engage their BCR and induce proper B cell activation for secondary remodeling events. Within the germinal center (GC), B cells undergo to two major genetic rearrangements initiated by AID (78): SHM and CSR at *IgH* loci whereas only SHM occurs at *IgL* loci. By inducing frequent point mutations into the variable regions of *Ig* loci, SHM is a driving force for antibody affinity maturation. CSR results in *µ* heavy chain replacement by another subtype (IgG, IgA or IgE) after initiating DNA double strand breaks and recombination. In addition, locus suicide recombination (LSR) is another rearrangement which leads to deletion of all *IgH* constant genes and therefore induces B cell death by abrogating surface B cell receptor expression (1). In parallel to such secondary remodeling events, activated B cells differentiate into antibody secreting plasma cel or memory B cells (73).

### IgH Nuclear Positioning and Loops in Mature Naive and Activated B Cells

**Figure 4:**
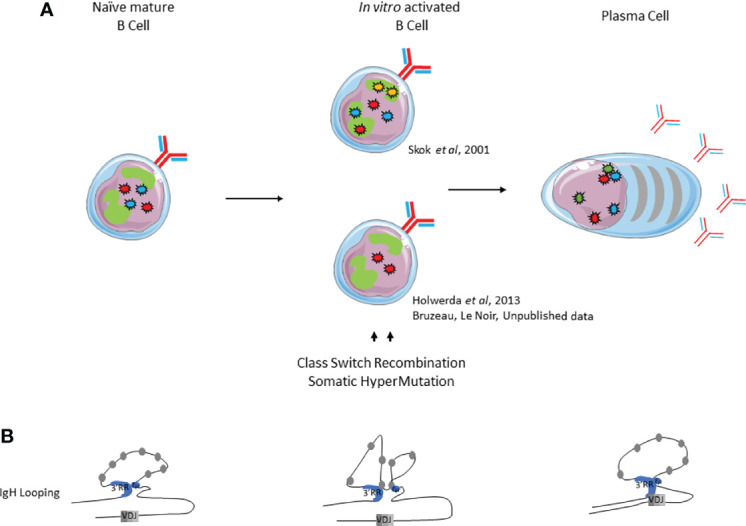
Immunoglobulin loci positioning and conformation within the nucleus during late B cell development. **(A)**. Nuclear positioning of IgH, Igk and IgJ chains. **(B)**. *IgH* looping. *IgH* alleles are represented by red stars, *Igκ* alleles by blue stars*, Igλ* alleles by yellow stars and *J chain* alleles by green stars.

Mature B cell differentiation and its accompanying late genetic remodeling events are also characterized by changes in nuclear organization and chromatin loops. Pioneering studies used 3D-FISH to show that *IgH* alleles were not located in similar positions within nuclei of resting naive and activated B cells. At the resting mature B cell stage, both *IgH* alleles are localized in euchromatin whereas their respective nuclear position upon *in vitro* activation is still debated. According to Skok’s lab, the *IgH* allele colocalized with PCH and replicated later, suggesting that this allele is the unproductive allele (72) (**Figure 4**). These elements imply that the non-productive *IgH* allele is “tagged and maintained as excluded” by nuclear location. Although when using the same 3D-FISH approach in stimulated cells, the De Latt group showed that both *IgH* loci are sitting in an active compartment (79) (**Figure 4A**). This observation is in agreement with another study in which *IgH* alleles relocalized to the nuclear periphery in proliferating splenic B cells (80). By using mouse models carrying IgM of “a” and “b” allotypes (respectively from C57Bl6 and SV129 backgrounds), Holwerda and colleagues also showed, by 4C-Seq, that tardive replication in splenic B cells was lymphoid specific but independent of nuclear location and topology of *Ig* loci (79). The fact that allelic exclusion might not be driven by nuclear location is in agreement with previous studies showing *IgH* biallelic expression in mature B cells (58, 81). Evidence for loop formation was first provided by the Kenter group that described high frequencies of interaction between *Eµ* and *3’RR* in mature resting B cells. Loop conformation within *IgH* changes upon *in vitro* stimulation by lipopolysaccharide (LPS) with or without interleukin (IL-4), which respectively induce CSR mostly towards IgG3 and IgG1; by acquiring additional contact between previously interacting enhancers and acceptor switch region (*Sγ_3_* or *Sγ_1_*) involved in CSR (75) (**Figure 4B**). The advantage of this architectural scaffolding, promoting synapsis between *S* regions, has been mechanistically demonstrated to facilitate CSR by the loop extrusion mechanism (74, 82). Briefly, cohesin is loaded at the *3’RR* end and the extrusion mechanism bring together *3’RR* and *Eµ* regions. An additional internal loop is further formed to juxtapose the transcriptionally active *S* regions. This particular conformation first allows AID recruitment at both *Sµ* and *S* acceptor regions to induce DSB and second maintains *S* regions together for ligation by the NHEJ pathway [for review (83)].

### Igκ Nuclear Positioning in Mature Naive and Activated B Cells

Similar observations can be made for *IgL* loci (**Figure 4**). *Igκ* monoallelic positioning in PCH is often observed in activated splenic B cells and this PCH-localized allele replicates later compared to the other one (64, 84). The current model proposes that this monoallelic positioning drives both light chain allelic and isotypic exclusion. Effectively, both *Igλ* alleles are located in repressive areas in the nuclear periphery of *in vitro* stimulated murine B cells, which mostly express *Igκ*.When *Igλ* expression is forced, one *Igλ* allele is recruited to a central permissive compartment within the nucleus and both *Igk* alleles are repositioned to the nuclear periphery, thus inhibiting *Igκ* expression (72). Moreover, colocalization of *Igκ* with *IgH* loci is observed more frequently upon LPS stimulation of B cells (65).

### Ig Nuclear Positioning and Chromosome Looping in Plasma Cells

The intense antibody secreting function of plasma cells requires high levels of immunoglobulin gene transcription. For this purpose, nuclear organization can now be considered as one important level of regulation as described by Garrard and colleagues (65). Although located on three different chromosomes, *Ig* genes (*IgH*, *Ig*κ and *Igλ*) in plasma cells often undergo physical clustering by forming pairs or triplets. Such clusters, often composed of functional alleles, preferentially localize within the same transcription factory near the nuclear periphery (**Figure 4A**). In this study, ChIP-3C-seq experiments performed with anti-RNA PolII antibodies indicate that physical interactions within transcription factories are mediated by *Ig* gene enhancers: *cEµ* and *3’RR* for *IgH*, *Eiκ* and *3’Eκ* for *Ig*κ loci. Nuclear location also facilitates transport of *Ig* transcripts from the nucleus towards the endoplasmic reticulum (65). Moreover, 3C experiments performed by Birshtein’s group underscored the importance of *IgH* chromatin conformation in a plasma cell line. In this study, authors showed that physical interactions between *V_H_* genes and the *3’RR* were involved in efficient *IgH* transcription, heavy chain expression and ensuing antibody synthesis (85). All of these elements demonstrate that nuclear organization contributes favorably to massive antibody synthesis (65).

### Role of Ig Enhancers on B Cell Nuclear Organization and Loops (**Table 1**)

While globally less documented, the implication of *Ig* gene enhancers in B cell nuclear organization (*Ig* positioning and loop formation) has been proposed in the context of the first-described *IgH* locus loop bringing the *3’RR* and *Eµ* regions into close contact before CSR. Upon LPS-stimulation, this configuration acquires additional contact between the acceptor *S* region involved in CSR and previous interacting *IgH* enhancers (75). In plasma cells, *Ig* loci “coordination” seems to be mediated by the *3’E*κ regulatory element since its deletion leads to a decrease in cohabitation of all *Ig* loci with decreases in *IgH-Igk*, *IgH-IgJ* and *Igk-IgJ* communication. Likewise, nuclear localization of these loci seems to be modified with the relocation of *IgH*, *Igk* and *IgJ* alleles further from the nuclear periphery in *3’Eκ* ^-/-^ plasma cells compared to *wt* plasma cells. Together, these results suggest that interactions between Ig genes (including interaction between *IgH* and *IgJ*) are mediated by *Igk*, especially by 3’Ek enhancer. Moreover, relocation and mis-cohabitation of *Ig* loci correlate with decreased transcription of each *Ig* gene, as observed in *3’Eκ* ^-/-^ plasma cells in comparison with *wt* cells (65).

*IgH* positioning within the nucleus might also be supported by the *3’RR*. Complete *3’RR* deletion leads to an increase in distance between both *IgH* loci in activated B cells (86). The pioneering study by Kenter’s group showed that *IgH* looping requires an intact *3’RR*. This statement is supported by the model devoid of its two last enhancers, *hs3b* and *hs4*, that led to a decrease in interactions between *3’RR* and *Eµ* regions in resting and LPS ± IL4 stimulated splenic B cells (75). In contrast, partial deletion of *3’CBEs* (*hs5 to 7*) does not impair loop formation at resting and activated B cell stages (26). However, Alt’s group reported that total *3’CBEs* deletion of led to significant decreases in interactions between *Eµ* and targeted switch regions in activated B cells (25). Deletion of the *cEµ* enhancer seems to have no impact on interactions between *3’RR* and *Eµ* regions since the contact frequencies in *cEµ* KO B cells are comparable to *wt* B cells (75). Changes in *IgH* locus conformation mediated by interactions between promoters and enhancers, could impair CSR by limiting the activity of promoters located upstream from each constant gene, and therefore, their ability to initiate prerequisite germline transcription (87).

## Discussion

Nuclear positioning and chromosome looping have clearly been shown to display a functional role in early developing B cells and in B cells in general. Beyond some discrepancies, studies overall showed that positioning and conformation of *Ig* loci play a major role in ordered H and L chains rearrangements. Furthermore, at such early stages, nuclear repositioning of *Ig* loci to PCH is moreover integral to allelic exclusion mechanism while chromosome looping optimizes antibody repertoire constitution. Regulatory regions of *Ig* gene loci largely participate in positioning and looping. At both *IgH* and *Igk, Eµ* and *3’RR* as well as *Cer* elements are essential for the proper loop formation in their respective loci. In contrast, *Igk Sis* element is supposed to ensure crosstalk between *IgH* and *Igk* loci since influencing *cis-* as well as *trans-*positioning. Similarly, *3’Eκ* also promotes, at early stages, temporal association between *IgH* and *Igκ*.

In contrast, in mature B cells, the role of *Ig* loci position within the nucleus remains more elusive since studies unveil various differences. Nevertheless, *IgH* chromosome looping has been distinctly shown to play a role in CSR, especially by bringing donor *Sµ* and acceptor switch regions in close proximity to optimize switch recombination events. During SHM, primary transcription of the *V* exon, potentially in both sense and antisense direction, is an important prerequisite for AID-induced mutations. In KO mouse models in which the *3’RR*palindromic structure is disrupted, the observed SHM defects correlate to a decrease in *V_H_* primary transcription (29, 35, 36). As transcription is an essential step to initiate loop extrusion prior CSR, establishing a link between SHM and loop extrusion would be of critical interest. In addition, the function of ncRNA in chromosome topology during CSR (88) and SHM (89) has been elucidated. In plasma cells, only few studies reported that *Ig* loci are colocalized to transcription factories, surely to improve antibody production. Similarly, the role of *Ig* loci conformation throughout terminal B cell development is also of major interest.

*IgH* locus positioning within the nucleus during CSR seems to be important for legitimate maintenance. While *IgH* nuclear location in close proximity to an oncogene is a contributing factor for translocation (90); its distal 3’RR enhancers *hs3b* and *hs4* have be shown to be only involved in oncogene activation but not in translocation (91). Given that AID activity initiates DNA modifications, it is mandatory that *IgH* rearrangements be tightly regulated to avoid any mutations or translocations within oncogenes (so called “AID off targets”). Several AID off target genes (*ie*: *Pax5*, *Il4ra* and *Inf8*) lie in close proximity to *IgH* during CSR and altogether are located in a chromosomal territory containing a high AID concentration (90). As a consequence, the non-random positioning and conformation of *Ig* loci could be widely considered as critical to maintain B cell genome integrity. In this way, their respective regulatory elements could play the role of B cell genome guardians thereby avoiding illegitimate events that contribute to lymphomagenesis.

## Author Contributions

CB wrote the manuscript and prepared the figures. SN wrote the manuscript. EP provided critical feedback. All authors contributed to the article and approved the submitted version.

## Funding

CB was supported by a Ph.D. fellowship of the french Ministère de l’Enseignement Supérieur, de la Recherche et de l’Innovation. ANR-21-CE15-0001-01.

## Conflict of Interest

The authors declare that the research was conducted in the absence of any commercial or financial relationships that could be construed as a potential conflict of interest.

## Publisher’s Note

All claims expressed in this article are solely those of the authors and do not necessarily represent those of their affiliated organizations, or those of the publisher, the editors and the reviewers. Any product that may be evaluated in this article, or claim that may be made by its manufacturer, is not guaranteed or endorsed by the publisher.
